# Outcomes and revision rates after antegrade continence enema (ACE) appendicostomy in a single-center LMIC paediatric cohort

**DOI:** 10.1007/s00383-026-06533-0

**Published:** 2026-07-12

**Authors:** C. Campilongo, E. Trovalusci, L. Hartford, C. Bebington, C. Westgarth-Taylor, Giulia Brisighelli

**Affiliations:** 1 Pediatric Surgery Unit, Department of Women’s and Children’s Health, Padua University Hospital, Padua, Italy; 2 Department of Pediatric Surgery, School of Clinical Medicine, Faculty of Health Sciences, University of the Witwatersrand, Johannesburg, South Africa; 3 Johannesburg Pediatric Colorectal Clinic, Chris Hani Baragwanath Academic Hospital, Chris Hani Road, Diephloof, Soweto, Johannesburg, South Africa; 4 Pediatric Colorectal and Urogenital Program, Baylor College of Medicine, Houston, TX USA

**Keywords:** Anorectal malformation, Antegrade continence enemas, Appendicostomy, Low- and middle-income countries, Malone, Neurogenic bowel

## Abstract

**Purpose:**

Antegrade continence enema (ACE) appendicostomy is widely used in children with complex colorectal and neurogenic bowel conditions, yet data from resource-constrained settings remain limited. We aimed to describe the experience of a single-center pediatric cohort in a low-middle-income country (LMIC).

**Methods:**

A retrospective review of all ACE procedures performed between 2012 and 2026 was conducted. Demographics, diagnoses, indications, complications, surgeries, and functional outcomes (assessed with Milan Bowel Function Questionnaire) were collected from clinical and operative records. Descriptive statistics were applied.

**Results:**

Forty-five children (80% male) underwent ACE procedures, with a median age of 8.3 years. Most had appendicostomy; two required cecostomy intraoperatively. The most common diagnosis was anorectal malformation (*n* = 33, 73.3%), followed by neurogenic bowel (*n* = 7, 15.5%). Eight patients (17.7%) underwent a concomitant Mitrofanoff procedure. The main complication was skin-level stricture (*n* = 17, 37.8%), representing the only indication for surgical revision (*n* = 15, 33.3%), with a median time to revision of 5.5 months. Nearly all patients achieved clinical success, with high satisfaction reported on the questionnaire.

**Conclusion:**

ACE appendicostomy is an effective and satisfactory option in LMICs, with outcomes comparable to high-income settings, supported by careful patient selection and ongoing quality improvement efforts.

## Introduction

 Constipation and faecal incontinence are common conditions in the pediatric population and are frequently encountered in surgical patients following reconstruction for anorectal malformations (ARMs) and Hirschsprung disease (HD), as well as in children with neurogenic bowel disorders and severe functional constipation [1–4]. Although most patients can be effectively managed with dietary modifications or stimulant laxatives, a subset fails to respond to these conservative approaches and requires mechanical colonic washout to achieve social continence [1].

Bowel washouts are an effective therapeutic option, but they can be associated with poor adherence, caregiver burden, and restrictions on daily activities. Antegrade continence enemas (ACE) offer an alternative approach, allowing antegrade instillation of fluid into the proximal colon to achieve complete colonic evacuation, thereby preventing stool impaction and overflow faecal incontinence. This treatment is associated with high levels of patient and caregiver satisfaction and has been shown to improve bowel function and quality of life [5, 6].

The first ACE procedure was described by Malone in 1990 and involves the use of the appendix to create a catheterizable conduit between the abdominal wall and the cecum, facilitating colonic irrigation [7]. Subsequently, the creation of a conduit through cecal tubularization was introduced for patients with an absent or unusable appendix [8, 9]. In 1996, Chait et al. described the percutaneous placement of a cecostomy tube, providing a minimally invasive alternative to appendicostomy [10].

ACE is now widely used in children with complex colorectal and neurogenic bowel conditions. However, outcome data from resource-constrained settings remain limited. Although South Africa is classified as an upper-middle-income country, Chris Hani Baragwanath Academic Hospital (Soweto, Johannesburg - South Africa) predominantly serves children from socioeconomically disadvantaged communities, with constraints typical of low- and middle-income settings. In this context, careful patient selection is essential, and socioeconomic constraints, limited resources, suboptimal home care, and poor continuity of follow-up may contribute to outcomes that differ from those reported in high-income countries (HICs) [11, 12].

This study aimed to describe the indications, outcomes, and revision rates associated with ACE appendicostomy in a single-center pediatric cohort cared for under resource-constrained conditions, with the goal of informing local quality-improvement efforts.

## Methods

### Study design

We conducted a retrospective review of all paediatric patients who underwent ACE conduit creation or surgical revision at Chris Hani Baragwanath Academic Hospital between January 2012 and March 2026. Electronic medical records, including operative notes, clinic notes, and bowel function assessments, were reviewed to extract demographic and clinical data of patients.

The primary objective was to describe indications, management, and the incidence and characteristics of postoperative complications and reinterventions in this cohort.

A secondary objective was to evaluate bowel management outcomes, focusing on social continence and patient-reported bowel function in daily life. Bowel function was assessed clinically and, in a subset of patients, using a structured questionnaire adapted from the Milan Bowel Function Questionnaire previously used at our institution to evaluate continence and bowel habits in children with anorectal malformations. The tool explores stool frequency, soiling, faecal incontinence, need for bowel management, and the impact of bowel dysfunction on daily activities and social participation. For the present study, items related to stool accidents, regularity of bowel movements, and social impact were evaluated [13].

Treatment success (social continence) was defined as at least one bowel movement per day and no more than one stool accident per week.

Continuous variables were summarised using descriptive statistics, and categorical variables were reported as frequencies and percentages.

## Practice patterns

At Chris Hani Baragwanath Academic Hospital, ACE procedures are offered only to carefully selected patients within a structured bowel management programme. Children must first achieve effective social continence with retrograde enema regimen before antegrade access is considered, and social and logistical factors (type of housing and ablution facilities, ability to adhere to daily washouts) are taken into account and incorporated into decision-making. Additional urinary and colorectal reconstructive needs are evaluated prior to operative planning, including the need for a concomitant Mitrofanoff channel.

**Fig. 1:**
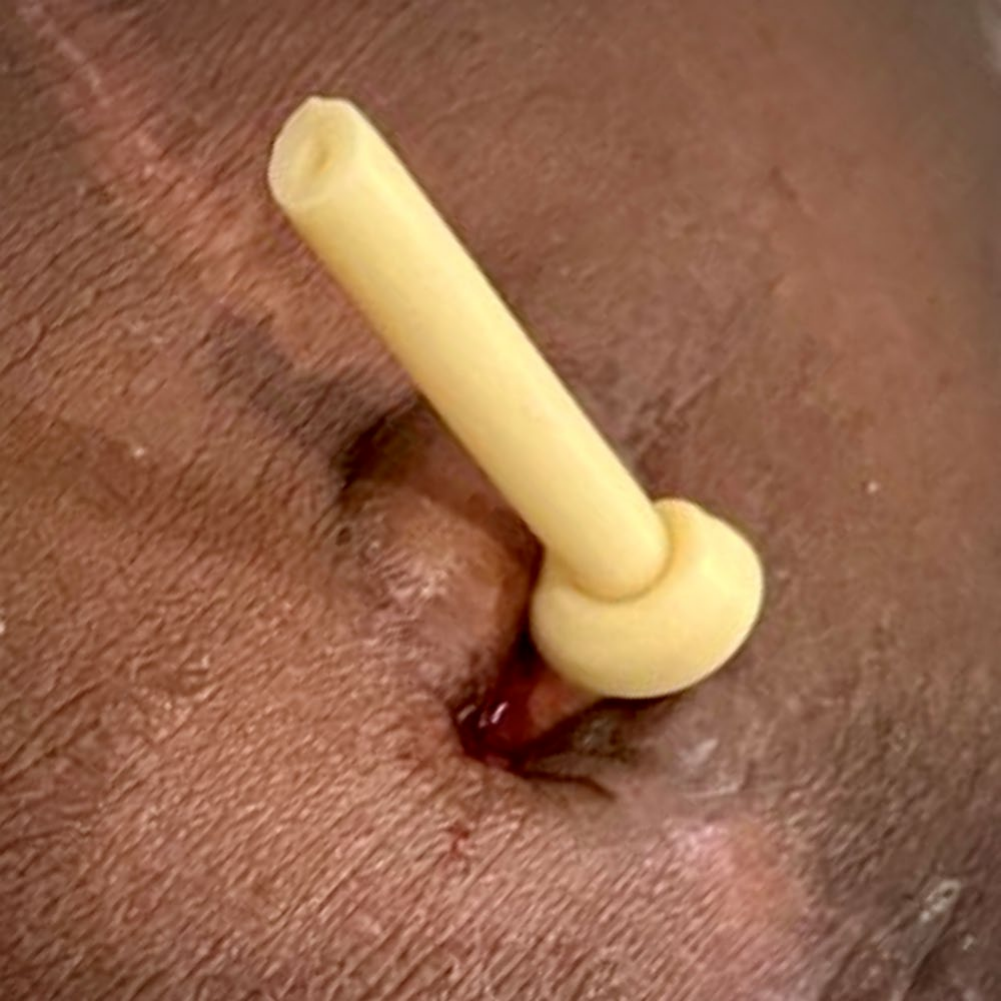
Alternative ACE stopper

An open surgical approach was used for both appendicostomy or neo-appendicostomy. Channels were usually brought out at the umbilicus or in the right or left lower quadrant, depending on patient anatomy and laterality, or the need of a Mitrofanoff channel. In female patients with anatomy compatible with future pregnancy, the ACE stoma is usually brought out in the right iliac fossa to minimise the risk of inadvertent injury to the conduit in case of a future need for caesarean section incisions. At the skin, a Y-V anastomosis was fashioned between the appendiceal mucosa and the skin to create a cosmetically hidden ACE stoma. When a simultaneous Mitrofanoff was required and the appendix was of adequate length (approximately 6–7 cm), a split-appendix technique was used to create separate channels for the ACE and Mitrofanoff, with the Mitrofanoff usually placed at the umbilicus and the ACE in the right lower quadrant. An anti-reflux, valve-like mechanism is created by plicating the cecum around the proximal appendix. At the end of the procedure, an 8 French Foley catheter is placed in the tract, the balloon is inflated and the catheter is secured to the abdominal wall and kept in place for six weeks. Antegrade flushes are initiated on day one post-ACE fashioning through the Foley catheter. After six weeks, the patient is seen in the clinic and the catheter is removed while patients and caregiver are instructed on how to catheterize the channel. An ACE stopper is given to the family and kept in place, in between catheterizations, for at least six months. To note, dedicated ACE stoppers or semi-permanent devices, such as the Mini-ACE™are no longer routinely available in South Africa, and import costs are prohibitive; therefore, in most cases a knotted rubber tube/Foley catheter is used as an alternative (Fig. 1).

In patients in whom an ACE conduit was not feasible because of a short appendix in the context of a simultaneous Mitrofanoff requirement, cecostomies were created using an open approach. The caecum was mobilised as needed and secured with reabsorbable stitches to the anterior abdominal wall; a catheterizable tract was then established, and a Mic-Key button was typically inserted into the caecum as an alternative to a Chait tube, which is rarely available in our setting.

## Results

### Patient characteristics, indications and procedures (Table 1)

**Table 1:** Baseline patients characteristics, indications and procedures

Patients characteristics	
Male sex, n (%)	36 (80%)
Accommodation characteristics	
Formal house, n (%)	27/34 (79.4%)
Informal house, n (%)	7/34 (20.6%)
Sanitation facilities characteristics	
Inside toilet, n (%)	16/34 (47.1%)
Outside yard flushing toilet, n (%)	10/34 (29.4%)
Outside yard pit latrine, n (%)	8/34 (23.5%)
Age at ACE creation (years)	
Median (IQR)	8,3 (6.7,12.3)
Underlying diagnosis	
ARM, n (%)	33 (73.3%)
Bowel neurogenic disorders, n (%)	7 (15.6%)
HD, n (%)	3 (6.7%)
Perineal trauma, n (%)	1 (2.2%)
Functional constipation, n (%)	1(2.2%)
ARM type	
Bladder neck fistula, n (%)	4/21 (19%)
Prostatic fistula, n (%)	2/21 (9.5%)
Bulbar fistula, n (%)	3/21 (14.3%)
Imperforate anus without fistula, n (%)	3/21 (14.3%)
Vestibular fistula, n (%)	2/21 (9.5%)
Rectal atresia, n (%)	1/21 (4.8%)
Cloaca, n (%)	2/21 (9.5%)
Cloaca exstrophy, n (%)	4/21 (19%)
Spinal and/or sacral defects among ARM patients	
No defects, n (%)	19/28 (67.9%)
Spinal only, n (%)	3/28 (10.7%)
Sacral only, n (%)	2/28 (7.1%)
Spinal + Sacral, n (%)	4/28 (14.3%)
Type of ACE channel	
Appendicostomy, n (%)	43 (95.6%)
Cecostomy, n (%)	2 (4.4%)
Procedure performed	
Appendicostomy only, n (%)	32 (66.7%)
Cecostomy only, n (%)	0
Appendicostomy + Mitrofanoff, n (%)	7 (15.6%)
Cecostomy + Mitrofanoff, n (%)	1 (2.2%)
Appendicostomy + others procedures, n (%)	4 (8.9%)
Cecostomy + others procedures, n (%)	1 (2.2%)

Forty-five children underwent ACE during the study period and were included in the analysis; three (6.6%) had undergone a previous ACE before 2012, but were included as they required redo appendicostomy during the study period. The majority of patients were male (36/45, 80%). Age at ACE creation was available for 36 patients (80%), with a median age of 8.3 years (IQR 6.7, 12.3). Fecal incontinence was the most common indication for ACE (44/45, 97.7%). Underlying diagnoses, housing/sanitation characteristics, type of appendicostomy fashioned, and concomitant procedures are summarised in Table 1.

To note, one patient with HD had trisomy 21, and the patient with functional constipation had developmental delay, not allowing retrograde washouts.

## Outcomes (Table 2)

**Table 2:** Complications and revisions

Follow-up duration (months)	
Total, Median (IQR)	51 (8, 91.2)
ARM, Median (IQR)	56 (11, 103)
Bowel neurogenic disorders, Median (IQR)	8 (6.5, 41.5)
HD, Median (IQR)	97 (93.5, 100.5)
Perineal trauma	12
Functional constipation	65
Complications	
Stricture, n (%)	17 (37.8%)
Wound infection, n (%)	2 (4.4%)
Leakage, n (%)	0
Prolapse, n (%)	0
Revisions	
Total, n (%)	15/45 (33.3%)
ARM, n (%)	9/33 (27.3%)
Bowel neurogenic disorders, n (%)	3/7 (42.9%)
HD, n (%)	3/3 (100%)
Perineal trauma, n (%)	0
Functional constipation, n (%)	0
Continued use of ACE at last follow-up	
Total, n (%)	29/35 (82.9%)
ARM, n (%)	21/24 (87.5%)
Bowel neurogenic disorders, n, (%)	7/7 (100%)
HD, n (%)	1/2 (50%)
Perineal trauma, n (%)	0/1
Functional constipation, n (%)	0/1

Outcome data from operative notes were available for all 45 patients, while clinical follow-up data (clinic notes and/or questionnaire) were available for 35/45 (77.8%). The most common complication was stricture of the conduit in 17 patients (37.8%), which occurred at the skin level in 15/17 (88.2%) and in the mid-appendix in 2/17 patients (11.8%). Fifteen patients (33.3%) required at least one surgical revision; in all cases, the indication was stricture, and the median interval from index surgery to revision was 5.5 months (IQR 3.75, 18). In two patients with a split appendix ACE/Mitrofanoff, revision involved conversion of the ACE conduit to a cecostomy due to mid-appendiceal stricture. Wound sepsis occurred in 2 patients (4.4%) and was managed conservatively. No prolapse or stomal leakage was observed during follow-up.

Interestingly, one patient (2.2%) developed monthly bleeding of the appendicostomy, accompanied by abdominal pain, which was subsequently attributed to concomitant endometriosis with endometrioma. The diagnosis was prompted by the onset of these symptoms, and clinical improvement has been observed with progestinic therapy.

Ten patients (22.2%) were lost to follow-up. Among the remaining 35, 29 (82.9%) were still performing ACEs at last review. Of the 6 (17.1%) who were no longer performing ACEs, 3 ARM patients had improved sufficiently to transition to laxatives alone, 2 had strictured channels and were on retrograde washouts awaiting revision, and 1 with HD and trisomy 21 underwent an urgent laparotomy with total colectomy and ileostomy formation at another healthcare facility.

The washout regimen via ACE was documented for 22 patients (48.8%), with 16/22 (73%) performing daily washouts, 1/22 (4.5%) on alternate days, 3/22 (13.5%) twice weekly, and 2/22 (9%) once weekly.

The Milan Bowel Function Questionnaire was administered to 19 patients (42.2%) at a median follow-up of 46 months (IQR 19, 91) after ACE surgery. Eighteen patients (94.7%) reported no more than one stool accident per week and were classified as clean for stool, meeting the definition of social continence. Table 3 summarises responses to items most relevant to continence, bowel habit regularity, and social impact. Table 4 presents the frequency of soiling and incontinence according to the type of toilet facilities used.

**Table 3:** Key questionnaire outcomes

What is his/her frequency of defecation?	
Every other day to twice a day, *n* (%)	13 (68.42%)
More often, n (%)	5 (26.32%)
Less often, n (%)	1 (5.26%)
What is the frequency of soiling episodes?	
Never, n (%)	10 (52.63%)
One a month or less, n (%)	5 (26.32%)
One a week or less, n (%)	3 (15.79%)
More than once a week but not daily, n (%)	1 (5.26%)
Daily/more than once a day, n (%)	0
What is the frequency of incontinence episodes?	
Never, n (%)	13 (68.42%)
One a month or less, n (%)	3 (15.79%)
One a week or less, n (%)	1 (5.26%)
More than once a week but not daily, n (%)	1 (5.26%)
Daily/more than once a day, n (%)	1 (5.26%)
During physical activity, does your child have soiling/incontinence episodes?	
Never, n (%)	12 (63.16%)
Sometimes, n (%)	5 (26.32%)
Always, n (%)	2 (10.53%)
During gastroenteritis/diarrhea or while on antibiotics, does your child have soiling/incontinence episodes?	
Never, n (%)	11 (57.89%)
Sometimes, n (%)	5 (26.32%)
Always, n (%)	3 (15.79%)
Do you believe that your child’s bowel habits cause social problems?	
Never, n (%)	13 (68.42%)
Sometimes, n (%)	5 (26.32%)
Always, n (%)	1 (5.26%)
Do you believe that your child’s bowel habits:	
Are more or less constant, n (%)	10 (52.63%)
Vary a lot according to predictable reasons (hydration, diet change, no time to perform enema, antibiotic therapy), n (%)	8 (42.11%)
Vary a lot according to unpredictable reasons, n (%)	1 (5.26%)

**Table 4:** Outcomes by sanitation/housing category

	Inside toilet (*n* = 8)	Outside flushing toilet (*n* = 5)	Outside pit latrine (*n* = 5)
What is the frequency of soiling episodes?			
Never, *n* (%)	2 (25%)	3 (60%)	3 (60%)
One a month or less, *n* (%)	3 (37.5%)	2 (40%)	0
One a week or less, *n* (%)	2 (25%)	0	1 (20%)
More than once a week but not daily, *n*n (%)	1 (12.5%)	0	1 (20%)
Daily/more than once a day, *n* (%)	0	0	0
What is the frequency of incontinence episodes?			
Never, *n* (%)	4 (50%)	5 (100%)	3 (60%)
One a month or less, *n* (%)	3 (37.5%)	0	0
One a week or less, *n* (%)	0	0	1 (20%)
More than once a week but not daily, *n* (%)	0	0	1 (20%)
Daily/more than once a day, *n* (%)	1 (12.5%)	0	0

## Discussion

Antegrade continence enema is widely used in the management of fecal incontinence and intractable constipation, not only in functional disorders but also in patients with complex colorectal and neurogenic bowel conditions [1–4]. In our cohort, non-functional conditions predominated, with ARM representing the largest group, followed by neurogenic bowel. Fecal incontinence was the main indication, reflecting the central role of bowel management in long-term care of children with ARM, HD, and neurogenic bowel, in whom sphincter hypoplasia, impaired sensation and sacral/spinal anomalies frequently limit the potential for spontaneous continence [13, 14].

Despite the complexity of our population and the constraints of a public-sector LMIC environment, ACE appendicostomy proved durable and effective. The long-term adherence was high, and almost all respondents met our definition of social continence, with low rates of soiling and a limited reported impact on school and social functioning. These findings are consistent with series from HICs, which report success rates of approximately 60–90% in mixed colorectal and neurogenic cohorts [15].

Compared with high-income settings, children treated at our institution face distinct structural barriers: many live in informal housing, lack indoor toilets, travel long distances to care, and have limited access to consumables and medications [11, 12]. These realities directly influence bowel management choices. In our experience, retrograde enemas can be difficult to perform reliably in crowded households with shared sanitation, whereas an ACE often allows a quicker, more predictable washout that older children can perform with greater independence. This may partly explain why bowel outcomes were at least as good in patients using outdoor toilets or pit latrines as in those with indoor facilities. Similar findings were observed in a previous ARM cohort from our centre [16].

In this context we may apply a lower threshold for offering ACE than in some high-income centres, particularly when the child and caregiver struggle with retrograde washouts but are motivated to adhere to a structured bowel programme. The procedure can also serve as a temporary bridge: in our series, several ARM patients subsequently transitioned to laxatives, underscoring that ACE need not be permanent in all cases.

Resource constraints also shape technical decisions. Commercial ACE stoppers and devices such as the Mini-ACE™ and Chait Trapdoor™ cecostomy tubes are scarcely available and often unaffordable. To reduce stenosis risk we advise patients to maintain a semi-permanent stopper, but in practice this frequently consists of improvised solutions (e.g. knotted feeding tubes or Foley catheters, and in one case an old phone-charging cable) rather than dedicated devices [11]. This is far from ideal, but it reflects the necessity of pragmatic, locally feasible solutions when standard equipment is inaccessible.

Finally, careful siting of the appendicostomy is particularly important in our setting, where many women will later deliver in peripheral hospitals without paediatric surgical support. We routinely position the appendicostomy in the right iliac fossa in females with potential for future pregnancy to reduce the risk of inadvertent transection during caesarean section by surgeons unfamiliar with ACE channels and in the absence of comprehensive medical records.

Appendicostomy is our preferred technique. All procedures were performed via an open approach, so in our setting cecostomy did not offer a minimally invasive advantage, and, in our limited experience, it required more maintenance and tube exchanges. Literature from high-income settings suggests broadly comparable overall success for appendicostomy and cecostomy but highlights a risk of serious cecostomy-related complications such as tract disruption and peritonitis [1, 8]. In resource-constrained environments, reduced dependence on proprietary tubes and a lower need for routine device changes are substantial advantages, supporting our preference for appendicostomy, when feasible.

In our series, the most frequent complication requiring surgical revision was stenosis, affecting approximately one-third of patients, which is at the upper end of reported ranges. Published stenosis rates after appendicostomy vary widely (roughly 11–41%), with substantial heterogeneity in definitions and follow-up [1–3, 15, 17]. Our higher rate likely reflects both structural and behavioural factors: limited access to catheters and stoppers, difficulties in replacing lost devices, lower health literacy, and inconsistent follow-up all reduce adherence to regular catheterisation, which is essential to maintain patency [1, 4, 18, 19]. Technical factors, such as appendiceal vascularity, mucocutaneous tension, and concomitant fashioning of a Mitrofanoff could also contribute, but only a minority of patients with a split appendix developed stenosis, supporting the feasibility of this technique when carefully selected [8]. However, even with this burden, serious complications were not observed.

The Milan Bowel Function Questionnaire, adapted from a tool originally developed for ARM patients, supported the overall effectiveness of ACE in daily life: most respondents reported normal or near-normal stool frequency, minimal soiling or incontinence, and limited social restriction [13]. Questionnaire scores were slightly lower than the favourable outcomes documented during clinical follow-up. This may reflect selection bias (only ~ 50% of eligible patients responded), early administration in some cases before full optimisation of the ACE regimen, and potential language or health literacy barriers. In addition, the use of an ARM-focused instrument introduces some construct mismatch for other diagnoses, although analysis was limited to relevant items. Importantly, despite these limitations, questionnaire scores remained overall favourable, reinforcing the effectiveness of ACE in this population rather than suggesting suboptimal outcomes.

Limitations of this study are inherent to the retrospective design. Data completeness depended on the quality of clinical records, follow-up intervals were variable, and one-fifth of patients were lost to follow-up, raising the possibility of under-ascertainment of complications. Minor wound issues and leakage may have been managed locally or outside the formal health system and not captured in our dataset. Questionnaire data were available for only a subset of patients and were collected at varying time points. Finally, we used an ARM-derived questionnaire across a heterogeneous diagnostic group, focusing on items that were most relevant to our aims, but this approach does not replace a diagnosis-specific validated tool.

Strengths include a relatively long observation period, detailed operative and follow-up data from a dedicated colorectal programme, and the integration of clinical and patient-reported outcomes in a genuinely resource-constrained environment. To our knowledge, few series from LMICs have reported ACE outcomes with this depth of context [3].

## Conclusion

ACE appendicostomy is an effective and durable bowel management option for children with complex colorectal and neurogenic conditions in a public-sector LMIC setting. Careful patient selection, attention to channel positioning and pragmatic adaptation to limited access to devices and follow-up allowed us to achieve continence and satisfaction rates comparable to those reported in high-income countries, despite higher stenosis and revision rates. These findings highlight that, when integrated into a structured bowel management programme and tailored to patients’ social realities, ACE programmes can provide meaningful gains in continence, independence and quality of life, even in resource-constrained environments.

## Data Availability

Dataset supporting this manuscript is available only on request in order to protect patients’ privacy.

## Abbreviations

ACE: Antegrade continence enemas
ARMs: Anorectal malformations
HD: Hirschsprung disease
HICs: High income countries
LMICs: Low- and middle- income countries

## References

[1] Halleran DR, Vilanova-Sanchez A, Rentea RM et al (2019) A comparison of Malone appendicostomy and cecostomy for antegrade access as adjuncts to a bowel management program for patients with functional constipation or fecal incontinence. J Pediatr Surg 54(1):123–128. 10.1016/j.jpedsurg.2018.10.008 30361073 10.1016/j.jpedsurg.2018.10.008

[2] Lopez JJ, Svetanoff WJ, Bruns N et al (2022) Single institution review of Mini-ACE^®^ low-profile appendicostomy button for antegrade continence enema administration. J Pediatr Surg 57(10):359–364. 10.1016/j.jpedsurg.2021.12.016 35090714 10.1016/j.jpedsurg.2021.12.016

[3] Olsbø S, Kiserud SG, Telle Hoel A, Stensrud K, Bjørnland K (2025) Systematic Review of Reported Outcomes for Antegrade Continence Enema in Patients With Anorectal Malformation and Hirschsprung Disease. J Pediatr Surg 60(11):162634. 10.1016/j.jpedsurg.2025.162634 40907881 10.1016/j.jpedsurg.2025.162634

[4] Merritt AT, Evans LL, Cooper EH, Peña A, de La Torre L, Bischoff A (2025) Outcomes after appendicostomy and neo-appendicostomy in a single institution. Pediatr Surg Int 41(1):189 Published 2025 Jun 24. 10.1007/s00383-025-06099-3 40553246 10.1007/s00383-025-06099-3

[5] Har AF, Rescorla FJ, Croffie JM (2013) Quality of life in pediatric patients with unremitting constipation pre and post malone antegrade continence enema (mace) procedure. J Pediatr Surg 48(8):1733–1737. 10.1016/j.jpedsurg.2013.01.045 1016/j. jpeds urg. 2013. 01.045 10.1016/j.jpedsurg.2013.01.045 23932614

[6] Cushing CC, Martinez-Leo B, Bischoff A, Hall J et al (2016) Health-related quality of life and parental stress in children with fecal incontinence: a normative comparison. J Pediatr Gastroenterol Nutr 63(6):633–636. 10.1097/mpg0000000000 001201 27027905 10.1097/MPG.0000000000001201

[7] Malone PS, Ransley PG, Kiely EM (1990) Preliminary report: the antegrade continence enema. Lancet 336(8725):1217–1218 1978072 10.1016/0140-6736(90)92834-5

[8] Goddard GR, Rymeski B, Jenkins T, Mullapudi B, Dickie BH, Bischoff A et al (2019) A comparison of surgical complications after appendicostomy and neoappendicostomy in pediatric patients. J Pediatr Surg 54:1660e3. 10.1016/j.jpedsurg.2019.04.002 31036369 10.1016/j.jpedsurg.2019.04.002

[9] Jalles F, Xu TO, Elhalaby I, Badillo AT, Levitt MA, Varda BK (2025) How to avoid a Monti. J Pediatr Urol 21(5):1350–1352. 10.1016/j.jpurol.2025.05.027 40527638 10.1016/j.jpurol.2025.05.027

[10] Shandling B, Chait PG, Richards HF (1996) Percutaneous cecostomy: a new technique in the management of fecal incontinence. J Pediatr Surg 31(4):534–537 8801307 10.1016/s0022-3468(96)90490-x

[11] Brisighelli G, Etwire V, Lawal T, Arnold M, Westgarth-Taylor C (2020) Treating pediatric colorectal patients in low and middle income settings: Creative adaptation to the resources available. Semin Pediatr Surg 29(6):150989. 10.1016/j.sempedsurg.2020.150989 33288130 10.1016/j.sempedsurg.2020.150989

[12] Brisighelli G, Loveland J, Bebington C, Dyamara L, Ferrari G, Westgarth-Taylor C Do social circumstances dictate a change in the setup of an anorectal malformation clinic? J Pediatr Surg Published online March 2020:S0022346820302086. 10.1016/j.jpedsurg.2020.03.012 10.1016/j.jpedsurg.2020.03.012 32273115

[13] Brisighelli G, Macchini F, Consonni D, Di Cesare A, Morandi A, Leva E (2018) Continence after posterior sagittal anorectoplasty for anorectal malformations: comparison of different scores. J Pediatr Surg 53(9):1727–1733. 10.1016/j.jpedsurg.2017.12.020 29370894 10.1016/j.jpedsurg.2017.12.020

[14] Rice-Townsend SE, Nicassio L, Glazer D et al (2023) Fecal continence outcomes and potential disparities for patients with anorectal malformations treated at referral institutions for pediatric colorectal surgery. *Pediatr Surg Int*. ;39(1):157. Published 2023 Mar 23. 10.1007/s00383-023-05447-5 10.1007/s00383-023-05447-5 36952009

[15] Jonker CAL, van der Zande JMJ, Benninga MA et al (2025) Antegrade Continence Enemas for Pediatric Functional Constipation: A Systematic Review. J Pediatr Surg 60(1):161952. 10.1016/j.jpedsurg.2024.161952 39389879 10.1016/j.jpedsurg.2024.161952

[16] Del Re G, Morandi A, Bebington C, Westgarth-Taylor C, Leva E, Brisighelli G (2025) Bowel management outcomes in anorectal malformations: a comparative study between two referral centers in a high- and a low/middle-income country. Pediatr Surg Int 41(1):178. 10.1007/s00383-025-06078-8. Published 2025 Jun 19 40536550 10.1007/s00383-025-06078-8

[17] Xu T, Hanke R, Samuk I et al (2024) Treatment of Persistent Soiling in Hirschsprung Disease With Antegrade Continence Enemas. J Surg Res 302:411–419. 10.1016/j.jss.2024.07.061 39153363 10.1016/j.jss.2024.07.061

[18] Siminas S, Losty PD (2015) Current surgical management of pediatric idiopathic constipation: a systematic review of published studies. Ann Surg 262(6):925e33 25775070 10.1097/SLA.0000000000001191

[19] Carnaghan H, Johnson H, Eaton S et al (2012) Effectiveness of the antegrade colonic enema stopper at preventing stomal stenosis: long-term follow-up. Eur J Pediatr Surg 22(1):26–28. 10.1055/s-0031-1285874 22270962 10.1055/s-0031-1285874

